# Treatment success and its predictors as well as the complications of catheter ablation for atrial fibrillation in a high-volume centre

**DOI:** 10.1007/s10840-021-01011-0

**Published:** 2021-05-31

**Authors:** Anna Numminen, Tero Penttilä, Olli Arola, Jaakko Inkovaara, Niku Oksala, Heikki Mäkynen, Jussi Hernesniemi

**Affiliations:** 1 Faculty of Medicine and Health Technology, Tampere University, Tampere, Finland; 2 Tays Heart Hospital, Tampere University Hospital, Tampere, Finland; 3 Vascular Centre, Tampere University Hospital, Tampere, Finland

**Keywords:** Atrial fibrillation, Pulmonary vein isolation, Catheter ablation, Predictors, Complications, Redo

## Abstract

**Purpose:**

Catheter ablation for atrial fibrillation (AF) is a standard procedure for maintaining sinus rhythm. The aim of this study was to evaluate treatment success and its predictors and to provide quality control data on complications and redo operations in a centre with an initially a low but currently high annual volume.

**Methods:**

Data on patients (*n* = 1,253) treated with catheter ablation for AF in Tays Heart Hospital between January 2010 and May 2018 was evaluated (*n* = 1178 ablation-naïve patients and *n* = 1514 AF ablations). Comprehensive data on patient characteristics, treatment results, redo operations and complications were collected. Treatment success (maintenance of sinus rhythm at 1 year) was evaluated among patients residing within the hospital district (45% of the entire study population).

**Results:**

Treatment success was observed in approximately 62.9% of the ablation-naïve patients. Preoperative predictors of treatment success were paroxysmal AF type, previous use of antiarrhythmic drugs, left atrium diameter and age. The experience at the centre did not associate with the 1-year outcome. A relapse during the first 3-month blanking period was associated with a nine-fold risk of failure at 1 year (unadjusted OR 9.1, 95% CI 5.5–15.1, *p* < 0.001). The major complication rate was 4.5% (68/1514) with no deaths. Ten percent of the patients needed a redo procedure within the first year.

**Conclusions:**

Patient-related factors are the most significant predictors of treatment success. A relapse during a 3-month blanking period is associated with a very high risk of failure at 1 year.

## Introduction

Catheter ablation for atrial fibrillation (AF), similarly to antiarrhythmic drug (AAD) therapy, is generally an effective and relatively safe therapy aimed at restoring and maintaining sinus rhythm (SR) and, subsequently, also improving the quality of life [1–3]. However, the success rate of AF ablation varies considerably in published reports [4], as do the complication rates, especially in a comparison between high- and low-volume centres [5]. In order to be considered as a first-line therapy in any given centre, the success rate for catheter ablation should be acceptable, with a correspondingly low complication risk [1, 6]. Quality control is also necessary for the purposes of evaluating cost-efficiency [7].

In addition to centre size, other patient- or procedure-related factors have been identified as potential predictors of the maintenance of sinus rhythm after catheter ablation for AF. These factors include the patient’s age, atrial fibrillation type (paroxysmal vs persistent), freedom from early recurrence at 3 months after the procedure, as well as left atrial size, left atrial fibrosis, obesity and other underlying conditions [8–12]. In addition, there have been attempts to generate prediction models for AF recurrence after ablation, such as the APPLE score [13–15].

The purpose of this retrospective study was to examine the performance of a large centre performing catheter ablations for atrial fibrillation and to evaluate the prognostic value of several clinical variables as regards treatment success. This data adds to the cost-efficiency analysis of catheter ablation and to the evaluation of the prognostic value of previously identified risk factors for AF recurrence, providing quality control data from a large centre (Tays Heart Hospital) responsible for tertiary services for a district with over one million residents in Finland.

## Material and methods

### Study setting

This is a retrospective registry study on patients treated with catheter ablation for AF by means of pulmonary vein isolation (PVI) between 1 January 2010 and 15 May 2018 in Tays Heart Hospital. A dedicated electronic database for recording the details of invasive procedures (KARDIO) was used to identify patients who had undergone ablation for AF between 1 January 2010 and 15 May 2018 (*n* = 1253 patients with *n* = 1514 procedures). The study centre, Tays Heart Hospital, is a specialised tertiary care centre in Tampere (Finland). Ablations for atrial fibrillation were not frequently performed during the first years of the study period (*n* < 50 in 2010 and *n* = 107 in 2011), but since 2013, the annual number of procedures exceeded 200. In addition to patients residing in the hospital district itself (Pirkanmaa Hospital District), Tays Heart Hospital receives patients from other hospital districts, resulting in a catchment area of approximately 1 million inhabitants. Details of the study centre have been published earlier [16].

### Ablation procedures

Catheter ablations for atrial fibrillation were performed by following the clinical practice guidelines of the European Society of Cardiology [1]. The main target was the isolation of pulmonary veins (PVI) either by means of the radiofrequency (RF) technique (86.5% of all procedures, *n* = 1310/1514) or the cryoablation technique (13.5%). In redo-ablations (22.2%, *n* = 336/1514), the aim was the re-isolation of the pulmonary veins with the RF technique. PVI was always verified with a Lasso or Pentaray catheter in RF ablations and with an Achieve catheter in cryoablations. Additional linear ablations or structure modifications were performed infrequently and only when necessary. The cavo-tricuspid isthmus line (CTI) was ablated with the RF technique during the same procedure if there was clinical documentation of a typical atrial flutter (*n* = 284/1514, 18.8%). According to the data available, the Biosense Webster Carto 3 system with up-to-date catheters was the most frequently used 3D mapping system (94%) in RF ablations. A magnetic navigation system was used in 54% of the RF ablation procedures. The Medtronic cryoablation system with an Arctic Front second-generation balloon catheter was used in all cryoablation procedures. RF ablations were performed during the whole study period, as cryoablations commenced in October 2015.

### Use of oral anticoagulants and antiarrhythmic drugs

OAC therapy was started at least 3 weeks before and continued for at least 3 months after the procedure for patients not already on permanent OAC. Warfarin was used by 43% of the patients (INR target 2–3 and no periprocedural discontinuation). The use of direct oral anticoagulants (DOAC) became more prevalent during the observation (study) period, and bridging therapy with low-molecular-weight heparin (LMWH) was used until 2017. Thereafter (with the availability of idarusitsumab), the periprocedural OAC was dabigatran, according to the protocol adopted in the RE-CIRCUIT trial [17]. In brief, other DOACs were temporarily replaced with dabigatran for continuous periprocedural use (no additional bridging therapy) 3 weeks before the procedure. The switch back to the original DOAC was made 1 week after the procedure. If the CHA2DS2-VASc score was 2 or more, oral anticoagulation was continued permanently. In 43.7% (*n* = 515/1178) of ablation-naïve patients, AAD was continued for a short period of time (based on electronic medication lists). AADs were usually planned to be discontinued after three-month if there were no relapses.

### Data extraction

The KARDIO database is maintained and updated by physicians and nurses (during hospitalizations, different invasive procedures and outpatient visits), recording detailed periprocedural information on all procedures at the study centre. The data from the KARDIO registry was combined with data from the hospital district’s electronic patient record database and from a full-disclosure manual review of written hospital records. The combined data was stored in a single database containing all relevant disease-related phenotype information, procedure-related information, outcome information and mortality data (the MADDEC database). The structure of the MADDEC database has been previously described in more detail [16]. In brief, the collected data includes patient characteristics, prevalent comorbidities, parameters measured during a cardiac ultrasound examination and laboratory data. This data was completed, if available, with the information searched from written but electronically recorded primary and specialised care outpatient and hospital records. These included the year of first AF episode, more detailed cardiac ultrasound parameters, the AADs used, previous ablations, 3-month results, rehospitalisation and cardioversion rates 1 year after the procedure, 1-year results with detailed information about Holter results, as well as possible symptoms and late occurrence rates. Full outcome data was available for all complications.

Paroxysmal and persistent AF were defined according to ESC/EHRA definition (paroxysmal AF terminates spontaneously or with intervention within 7 days of onset and persistent atrial fibrillation is continuously sustained beyond 7 days, including episodes terminated by cardioversion after ≥ 7 days).

### Quality parameters

The following quality parameters were used: (1) proportion of patients with sinus rhythm (treatment success) at the 1-year control after the first AF ablation and (2) the number of major periprocedural complications per ablation. In addition, the proportion of patients needing a redo ablation during the whole follow-up time was evaluated.

The first quality parameter was assessed among patients residing within the hospital district (data available for 45% of all ablation-naïve patients, *n* = 532/1178). In this population, adequate 1-year control data was available for evaluating success or failure, using the hospital district’s specialised and primary care visit data (texts and other documentation, including ECG documents).

The endpoint (treatment success) was evaluated for the period of after a 3-month blanking period until 1 year from the procedure. The maintenance of normal sinus rhythm was assumed if there was no documentation of a relapse of atrial fibrillation or a flutter in written primary and specialised care patient records and recorded ECGs and, when complete 1-year control visit data was available (in 64% of cases, see below), there was no evidence of a relapse of atrial fibrillation or a flutter at the 1-year routine outpatient clinic visit (including normal ECG and 24-h Holter recordings). A relapse (i.e. failure) was recorded if atrial fibrillation or a flutter was reliably documented after a 3-month blanking period in the patient records (including ECGs) or at the 12-month outpatient clinic visit (including ECG and 24-h Holter), and/or if AADs had to be continued at the 1-year control and/or the patient was assigned to redo or AV-nodal ablation at or before 1-year outpatient clinic visit.

For the evaluation of treatment success, complete 1-year control data with 24-h Holter recording was available in 64% (*n* = 340/531) of the cases. In the remaining 36% of the cases, outcome data was based only on the existence or absence of documentation (text or ECG) of a relapse in patient records. For this reason, the results were further verified in a sensitivity analysis including only the subjects with full control visit data. The results were similar in both comparisons, and both results are presented.

For the second quality parameter (complications), a major complication was defined according to the *2017 HRS/EHRA/ECAS/APHRS/SOLAECE expert consensus statement on catheter and surgical ablation of atrial fibrillation* [18] as a complication that results in permanent injury or death, requires intervention for treatment, or prolongs or requires hospitalization for more than 48 h. Periprocedural complications were followed for up to 1 year after ablation for AF, although most of the complications (with exception of pulmonary vein stenosis) occurred immediately or were discovered during the first month after the operation. Data on redo operations and periprocedural complications were available and adequate for all patients (and procedures).

### Statistical analysis

Continuous variables were compared across groups with Student’s *t* test. The Chi-squared test was used to analyse the distribution of categorical variables. Multivariable regression analysis was applied to control for confounding factors. The association between centre experience and maintenance of normal sinus rhythm was tested by stratifying the whole study period into three periods with equal numbers of operations. The first period (January 2010–November 2013) was the longest because the annual volume of operations increased considerably during first 3 years (*n* = 36 in 2010, *n* = 106 in 2011 and *n* = 163 in 2012), after which it plateaued (*n* > 200 annually). The Kaplan–Meier method was used to estimate event rates for redo procedures. Competing events (deaths) occurring within 1 year from the procedure were excluded from the Kaplan–Meier analysis. All reported significance values are two-sided *p* values. A nominal *p* value of 0.05 was considered significant. To compare the results over time, the median time of the procedures was selected as a cut-off point. Statistical analyses were carried out with SPSS version 25.

## Results

### Baseline characteristics

Baseline characteristics are presented for all patients (*n* = 1253) undergoing their first ablation in the study centre during the study period (Table 1). The majority (94%) of the procedures were performed on ablation-naïve patients (*n* = 1178), and 68.2% of the patients were men. The remaining 6% of the patients (*n* = 75/1253) had undergone PVI in the study centre or elsewhere before the study period. The mean age of the population during the first operation in the study centre was 59 years (SD ± 9.4). The majority of the procedures were performed on patients with paroxysmal atrial fibrillation (62.9%, *n* = 749).

**Table 1:** Baseline characteristics of patients undergoing their first catheter ablation in Tays Heart Hospital between January 2010 and May 2018. The number of patients is reported first, followed by the valid percentage, except for continuous variables

Number of patients	1253
Age (years), mean ± SD	59.1 ± 9.37
Sex (males),	854 (68.2)
Number of previous atrial fibrillation ablations	
- None	1178 (94.0)
- One	62 (4.9)
- Two or more	13 (1.0)
Type of atrial fibrillation	
- Paroxysmal	749 (62.9)
- Persistent	424 (35.6)
- Long-standing persistent	18 (1.5)
Cryoenergy ablation	203 (16.2)
Radio frequency energy ablation	1050 (83.8)
Years since diagnosis, median ± IQR^*^	4 ± 6
Follow-up time (years), median ± IQR	3.71 ± 3.54
Body mass index (kg/m^2^), mean ± SD^*^	27.9 ± 5.02
Hypertension	518 (41.3)
Dyslipidemia	250 (20.5)
Coronary artery disease	41 (3.3)
Diabetes mellitus	93 (7.4)
Chronic heart failure	47 (3.8)
Previous transient ischaemic attack or stroke	55 (4.4)
Chronic obstructive pulmonary disease^*^	9 (0.7)
CHA_2_DS_2_-VASc ≤ 1	790 (63.0)
EHRA score 3 or more^*^	911 (96.8)
Antiarrhythmic drugs tested before ablation^*^	
- None	232 (23.0)
- Flecainide	467 (46.3)
- Dronedarone	82 (8.1)
- Amiodarone	107 (10.6)
- Multiple AADs	121 (12.0)
Anticoagulation*	
- Warfarin	278 (43.1)
- Dabigatran	291 (45.1)
- Abixaban	28 (4.3)
- Rivaroxaban	48 (7.4)
Left atrium (mm), mean ± SD	41.0 ± 4.81
Left ventricular ejection fraction (%), mean ± SD	62.3 ± 6.93
Left ventricle (mm), mean ± SD^*^	51.7 ± 5.02
Aortic stenosis^*^	3 (0.3)
Aortic regurgitation^*^	34 (3.3)
Mitral regurgitation^*^	78 (6.7)
Mechanical mitral valve or mitral valve repair^*^	7 (0.7)
Haemoglobin, mean ± SD^*^	145 ± 12.2
Glomerular filtration rate, mean ± SD	88.5 ± 12.5

^*^Data is missing for > 5% of the study population. *EHRA*, European Heart Rhythm Association; *SD*, standard deviation; *IQR*, interquartile range

### Treatment results

At 1 year, 62.9% (*n* = 334/531) of the patients in the hospital district undergoing their first ablation had successfully maintained sinus rhythm. One patient died during the first year of follow-up and was excluded from the analyses. In the sensitivity analysis including only subjects with full control visit data, normal sinus rhythm was observed in 61.8% (*n* = 210/340 of patients with 24-h Holter-verified control visit data on the maintenance of sinus rhythm).

Among all patients in the hospital district, significant preoperative predictors of the treatment result in univariate analyses were age, atrial fibrillation type, left atrial size, glomerular filtration rate (GFR), pre-existing hypertension and previous use of AADs (*p* < 0.05 for all in univariate analyses, see Tables 2 and 3). According to multivariable analysis, left atrial diameter and previous AAD use remained as significant and AF type as borderline significant predictors of treatment success (maintenance of normal sinus rhythm): OR 0.60 (95% CI 0.40–0.91, *p* = 0.016) for a 1-cm increase in left atrium diameter, OR 0.50 (0.313–0.808, *p* = 0.005) for previous use of AADs, and OR 1.49 (0.97–2.22, *p* = 0.051) for paroxysmal AF type. In this multivariable analysis, the association between age and treatment result became non-significant, but with a trend towards decreasing probability of treatment success with advancing age (OR 0.85 corresponding to one decade of life, with a 95% CI of 0.68–1.05, *p* = 0.127). GFR or pre-existing hypertension did not persist as an independent risk factor after adjusting for significant risk factors in multivariable analyses (*p* > 0.25 for both).

**Table 2:** Ablation-naïve patients treated in Tays Heart Hospital and residing in Pirkanmaa Region, stratified by catheter ablation procedure’s one-year outcome (*n* = 531)

	Sinus rhythm	Failure	*P* value
Age, mean (SD), year	58.9 (9.1)	60.7 (9.2)	0.033
Left atrial diameter, mean (SD), mm ^*^	40.0 (0.5)	41.4 (0.5)	0.002
Ejection fraction, mean (SD), % ^*^	62.0 (7.2)	62.2 (7.3)	0.692
Body mass index, mean (SD), kg/m^2 *^	27.7 (4.2)	28.1 (4.5)	0.380
Glomerular filtration rate, mean (SD), ml/min^*^	89.0 (12.7)	86.7 (12.3)	0.046
AAD			
Used before ablation (*n* = 403)	59.1% (238)	40.9% (165)	0.001
Not in use before ablation (*n* = 128)	75.0% (96)	25.0% (32)	
Atrial fibrillation type^*^			
Paroxysmal (*n* = 329)	67.2% (221)	32.8% (108)	0.005
Persistent (*n* = 166)	54.2% (90)	45.8% (76)	
Ablation type			
Radiofrequency ablation (*n* = 426)	61.3% (261)	38.7% (165)	0.117
Cryo (*n* = 105)	69.5% (73)	30.5% (32)	
Sex			
Male	64.0% (222)	36% (125)	0.481
Female	60.9% (112)	39.1% (72)	
Results over time			
First period (Jan 2010–Nov 2013)	60.6% (114)	39.4% (74)	0.305
Second period (Dec 2013–Feb 2016)	60.3% (91)	39.7% (60)	
Third period (Mar 2016–May 2018)	67.2% (129)	32.8% (63)	
Total (*n* = 531)	62.9% (334)	37.1% (197)	

^*^Data available for left atrial diameter in 525/531 (98.9%), for ejection fraction in 518/531 (97.6%), for BMI in 414/531 (78.0%), for GRF in 512/531 (96.4%) and for atrial fibrillation type in 495/531 (93.2%) of the cases

**Table 3:** Comorbidities and AF catheter ablation’s 1-year outcome

	All patients	Sinus rhythm	Failure	*P* value
Hypertension	44.4% (*n* = 236)	40.4% (*n* = 135)	51.3% (*n* = 101)	0.015
Obesity (BMI ≥ 30 kg/m^2^)^*^	28.7% (*n* = 119)	29.6% (*n* = 76)	27.4% (*n* = 43)	0.634
Diabetes	8.5% (*n* = 45)	8.1% (*n* = 27)	9.1% (*n* = 18)	0.674
History of heart failure episode	5.8% (*n* = 31)	5.7% (*n* = 19)	6.1% (*n* = 12)	0.848
EF ≤ 45% before ablation^*^	3.1% (*n* = 16)	3.1% (*n* = 10)	3.1% (*n* = 6)	0.971
Peripheral artery disease	6.2% (*n* = 33)	6.9% (*n* = 23)	5.1% (*n* = 10)	0.404
Ischaemic stroke	4.5% (*n* = 24)	5.1% (*n* = 17)	3.6% (*n* = 7)	0.410
Myocardial infarction^*^	3.0% (*n* = 15)	3.2% (*n* = 10)	2.6% (*n* = 5)	0.732
COPD^*^	1.8% (*n* = 9)	1.6% (*n* = 5)	2.1% (*n* = 4)	0.666
Mitral insufficiency^*^	9.8% (*n* = 46)	8.6% (*n* = 25)	11.7% (*n* = 21)	0.261

*BMI*, body mass index; *EF*, ejection fraction; *COPD*, chronic obstructive pulmonary disease

^*^Data available for ejection fraction in 518/531 (97.6%), for myocardial infarction in 506/531 (95.3%), for COPD in 506/531 (95.3%), for mitral insufficiency in 417/531 (88.7%) and for BMI in 414/531 (78.0%) of the cases

Ablation method, patient’s sex, left ventricular ejection fraction, body mass index or other pre-existing comorbidities did not significantly associate with treatment success in univariate analyses (Table 2), or in multivariable analyses adjusted with AAD use, left atrial size and atrial fibrillation type (data not shown). The APPLE score, which is determined based on age, AF type, GFR, left atrial size and left ventricular ejection fraction (one point each assigned for age > 65, persistent AF type, GFR < 60 ml/min, left atrial dimeter ≥ 43 mm and LVEF < 50%), associated significantly with treatment success: 74.5% (*n* = 188) of the patients with no risk points had maintained sinus rhythm at 1 year, compared to 59.4% (*n* = 123), 53.7% (*n* = 58), and 46.4% (*n* = 13) of patients with one, two, and three or more points, respectively (*p* < 0.001). Although the results showed a tendency towards improvement over time, centre experience was not significantly associated with treatment results (Table 2).

### The association between treatment outcome during the blanking period and the 1-year outcome

Control visit data for the blanking period (routine 3-month control visit data) was available for 81% of the patients with also one-year outcome data (*n* = 435/531). After the operation, the maintenance of normal sinus rhythm during the first 3-month blanking period (no symptoms at the clinical control, no ECG documentation of AF and SR in 24-h recording) also often led to sinus rhythm at 1 year (78.7%, *n* = 214/272). In contrast, a probable failure (patient-reported symptoms at the control visit but no documented failure during the blanking period) or definite failure (failure documented with ECG or verified by 24-h Holter at the 3-month routine control visit) resulted in the maintenance of sinus rhythm in only 44.2% (probable failure *n* = 23/52) and 28.8% (definite failure *n* = 32/111) of the cases. In univariate analysis, symptoms with no documentation of AF seemed to correspond to an approximately two-fold risk (OR 1.96, with 95% CI 0.99–3.89, *p* = 0.054) and documented failure associated with a nine-fold risk of failure at 1 year (OR 9.11, 95% CI 5.51–15.06, *p* < 0.001). After adjusting for other significant baseline predictors of failure (left atrium diameter and AAD use, both < 0.05), the results remained similar (OR 2.20, 95% CI 1.17–4.12, *p* = 0.014, and OR 10.12, 95% CI 6.24–16.40, *p* < 0.001).

### Complications

The overall complication rate for all 1514 procedures was 7.4%, with a 4.5% major complication rate. There were no statistically significant differences in major complication rates between men and women (4.2% vs. 5.1%, *p* = 0.476) or between patients with a BMI of less than 30 and those with a BMI of 30 or more (3.7%, *n* = 32, vs. 5.8%, *n* = 20, *p* = 0.110), nor was there a significant difference in the mean age between patients who suffered a complication (59.3 ± 9.4) and those who did not (60.2 ± 9.4, *p* = 0.449). Pericardial effusion (*n* = 32, 2.1%) and puncture site complications (2.0%) were the most common types of complications. Only three patients had an ischaemic stroke, defined as a major complication (0.2%), and no one died as a result of AF ablation. The occurrence of different types of complications is presented in Table 4.

**Table 4:** Periprocedural complications in 1514 catheter ablations for atrial fibrillation in Tays Heart Hospital between January 2010 and May 2018

Complication type	Minor *n*	%	Major *n*	%	Total *n*	%
Pericardial effusion						
Pericardial effusion, no drainage	5	0.3	11	0.7	16	1.1
Pericardial effusion, drainage	0	0.0	16	1.1	16	1.1
Pericarditis	5	0.3	5	0.3	10	0.7
Total	10	0.6	32	2.1	42	2.9
Bleeding complications						
Punct. site haematoma. no thrombin	6	0.4	5	0.3	11	0.7
Punct. site haematoma. thrombin	0	0.0	9	0.6	9	0.6
Punct. site bleeding, surg. intervention	0	0.0	7	0.5	7	0.5
Other punct. complication	2	0.1	0	0.0	2	0.1
Punct. site AV malformation	0	0.0	1	0.1	1	0.1
Punct. into aorta	0	0.0	3	0.2	3	0.2
Total	8	0.5	25	1.7	33	2.2
Thromboembolic complications						
Transient ischaemic attack	2	0.1	0	0.0	2	0.1
Ischaemic stroke	1	0.1	3	0.2	4	0.3
Total	3	0.2	3	0.2	6	0.4
Other complications						
Phrenic nerve palsy	3	0.2	2	0.1	5	0.3
Longer hospital stay (any reason)	9	0.6	0	0.0	9	0.6
Pulmonary vein stenosis	1	0.1	3	0.2	4	0.3
Shock	1	0.1	0	0.0	1	0.1
Pulmonary embolism	0	0.0	1	0.1	1	0.1
Tamponade and phrenic nerve palsy	0	0.0	1	0.1	1	0.1
Urinary tract infection	4	0.3	0	0.0	4	0.3
Urosepsis	1	0.1	0	0.0	1	0.1
Air embolism into coronary artery	4	0.3	0	0.0	4	0.3
Pleuritis	0	0.0	1	0.1	1	0.1
Total	23	1.7	8	0.6	31	2.3
TOTAL	44	2.9	68	4.5	112	7.4

### Redo operations

**Fig. 1:**
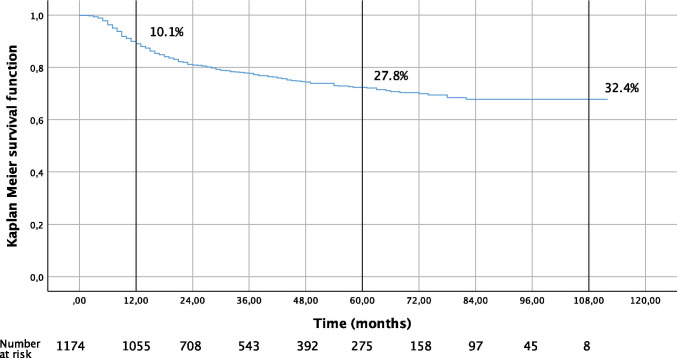
Kaplan–Meier presentation of redo procedures for patients going through their first pulmonary vein isolation

Of the patients who had their first AF ablation, 10.1% underwent a redo procedure during the first year of follow-up. In long-term follow-up, 32.4% of the patients needed a redo operation (Fig. 1). During the follow-up time (median of 3.7 years), only a minority (2.8%) underwent more than one redo operation (Table 5). Four patients died before the 1-year follow-up and were excluded from the analysis.

**Table 5:** Number of redo operations during a median follow-up time of 3.7 years in ablation-naïve patients

Number of redo operations	*N*	%
0	898	76.5
1	244	20.8
2	30	2.6
3	2	0.2
Total	1174	100

Four out of the 1178 patients undergoing their first ablation died during the first year of follow-up

## Discussion

The results of this retrospective registry study of patients undergoing catheter ablation for atrial fibrillation verify that, in a large-volume centre, sinus rhythm is successfully and safely achieved at 1 year in the majority of patients undergoing procedures for atrial fibrillation. Our results reaffirm the observations that the maintenance of sinus rhythm is associated with AF type and left atrium diameter, as well as the APPLE score, which comprises these two risk factors. Furthermore, according to our data, previous AAD use is associated with a significantly lower probability of success, and a failure during the first 3 months after the procedure is associated with an approximately nine-fold risk of failure at 1 year.

### Treatments results

A recent large retrospective trial with 5392 patients by Pallisgaard et al. [19] showed improved results between the periods of 2005–2006 and 2013–2014, with 55% and 69% 1-year success rates, respectively. Our study shows similar results, with a 63% overall success rate 1 year after ablation. Our results are also in line with recent prospective trials, which compared cryoablation to RFA [20] and visually guided laser balloon ablation to RFA [21], with 65% vs. 64% and 61% vs. 62% 1-year success rates, respectively. The variation in the results is understandable due to the different definitions of treatment success (i.e. maintenance of sinus rhythm) and differences in study methodology. In the prospective studies, the follow-up scheme utilised weekly transtelephonic ECG recordings, with a second ablation allowed during the 3-month blanking period, whereas the retrospective study by Palligaard et al. applied only a register-data-based endpoint.

In the present study, the definition of the maintenance of normal sinus rhythm was based on patient records, ECG documentation and a routine control visit data with complementary 24-h Holter recordings. Although the Holter recording was usually scheduled routinely at 1 year after the procedure to complement the control visit, this follow-up data was missing for 36% of the patients residing within the hospital district. The reasons for the missing follow-up Holter data mostly entailed of administrative reasons (lack of resources for routine recordings), or the control was implemented by means of a telephone interview without a complementary routine Holter recording or re-evaluation of possible failure with ECG. For some patients, a Holter recording was to be scheduled only in the event of symptom recurrence. In order to control for possible bias due to missing data, the outcome data was complemented by a full-disclosure review of outpatient and hospital visit records, and the results were verified by a sensitivity analysis of patients with Holter-verified evidence of the maintenance of sinus rhythm.

The results of our study confirm the finding that ablation results are superior in paroxysmal AF when compared to persistent AF (the single-procedure success rate was 67.2% for paroxysmal AF and 54.2% for persistent AF). In a meta-analysis by Ganesan et al. [4], the pooled single-procedure efficacy rate at 12 months was 66.6% for paroxysmal AF and 51.9% for non-paroxysmal AF. Furthermore, in line with previous large meta-analyses or prospective randomised studies [22, 23], we did not observe a significantly better success rate for cryoballoon ablation when compared to RFA, although there was a tendency towards obtaining better results with a cryoballoon (69.5% vs. 61.3%). This non-significant difference can be associated with differences in patient selection, and the analyses in the present study are not suitable for real head-to-head comparison. Similarly, we did not observe significant differences in treatment success and centre experience, although a trend towards better results was observed during the last 2 years of the observation period. In the present study centre, the procedures began to increase from a small annual number (< 50 procedures) to over 200 annual procedures after the first 3 years, but this rapid increase and subsequent plateau in the annual volume does not correspond to the results, which show slightly better results only after multiple years with over 200 annual procedures. Although the treatment results over time have been previously observed to improve in large multicentre studies [19, 24], our results do not directly confirm this observation.

Along with reaffirming the association between AF type and treatment success, we also observed a significant association between left atrial size and the APPLE score that comprises these two risk factors. Age, which is also included in the APPLE score, also showed a significant association with treatment success in univariate analyses but became non-significant in the multivariable analyses. Previous use of AADs was also associated with a lower probability of success in this population. Failed AAD therapy has been found to be a significant predictor for AF recurrence both after RFA and cryo AF ablations [9, 25, 26]. Higher risk of AF recurrence probably results from substrate modification and AF progression while drugs are being used, and from the fact that AADs are used more often by patients suffering from persistent AF.

Early recurrence (within three months) after AF ablation has been observed to be a strong predictor of a poor long-term result, with the results worsening with the lateness of the recurrence [27–30]. In the present study, we observed a 26% recurrence rate for confirmed AF, which corresponds to findings in previous studies [31], although it is somewhat lower than the observed pooled recurrence rate of 38% with variable time definitions for the blanking period. Besides varied blanking periods (from 2 days to most being 3 months), the difference is explained by differences in AF type and follow-up methods in different studies. In our registry, sinus rhythm at 1 year was observed only in 28.8% of the patients with definite early recurrence (within a 3-month blanking period). In clear contrast, a large proportion (78.7%) of the patients who were asymptomatic and did not have documented failure at 3 months were also free from AF at 1 year. The fact that treatment success at 1 year is highly likely if there is no spontaneous recurrence of symptoms or documented AF supports the notion that only symptom-initiated controls are necessary later on for patients with no recurrence during the first 3 months after the procedure.

### Complications

An international survey of radiofrequency catheter ablation procedures from 2010 reported a 4.5% incidence of major complications, including a 1.3% rate of cardiac tamponade, a 0.94% rate of stroke or TIA, a 0.04% rate of atrialesophageal fistula and a 0.15% rate of death. The major complication rate in our study was also 4.5%. Pericardial effusion (PE) has been regarded as a good quality indicator. PE was found in 2.1% of the 1514 operations performed at the study centre, and an intervention (drainage) was required in half of those cases (1.1% of all ablations). Our numbers correspond quite well with the results of a fairly recent retrospective German multicentre trial [5] and the recent CABANA trial [32], in which PE requiring an intervention was found in 0.9% and 0.8% of the cases, respectively. The overall complication rate of 7.4% seems relatively high but most likely reflects the fact that all possible minor complications (such as a 1-day prolonged hospital stay) were reliably included with no missing data. Only around 1.5% of the complications were potentially life threatening (including cardiac tamponade that needed intervention, ischaemic stroke and puncture to aorta). Overall, the present results confirm the good safety profile of catheter ablation for the treatment of atrial fibrillation.

### Redo procedures

Our 1-year redo rate was 10.1%, which corresponds well with recent large studies [23, 33]. During the mean follow-up time of 3.7 years, roughly one-third (32.4% at 8 years) of the previously ablation-naïve patients had a redo operation. In a prospective 10-year long-term follow-up study by Gaita et al. [34], 43% of the 255 patients had a redo procedure. Unfortunately, we do not have longer term follow-up data to report the need for redo procedures over a longer period.

### Gender inequality

Gender inequality has been shown in many studies. In a Canadian study [35] with 2438 patients receiving ablation for AF, despite comprising 42.9% of the general AF cohort, women represented only 26.5% of the ablation cohort. In our study, 31.8% (*n* = 399) of the patients were women. The inequality has been speculated to reflect the overall inequality in cardiac procedures between men and women and to result from women being more reluctant to pursue catheter ablation and from the higher complication rate in women who undergo an ablation procedure, to mention a few. The latter is not supported by our data: even though women had slightly more complications than men, 5.1% vs. 4.2%, this difference was not statistically significant. Adding to this, there was no difference between the sexes in 1-year treatment results.

### Limitations

Our study has several limitations. Firstly, the results are based on a retrospective search of patient records and databases, with obvious possible limitations concerning missing or invalid data. Many of the patients regarded as having maintained SR may have had silent periods of AF, which can be better observed in a prospective settings with active ECG of pulse measurements. Secondly, as opposed to the most traditional prospective study design, some antiarrhythmic medications were continued after the 3-month control if the patient had experienced some palpitations or minor symptoms possibly related to AF. However, AADs were usually discontinued at least a few months before the control at 1 year (with exception of eleven patients who had their AAD discontinued at 1-year control). Furthermore, if the patient had to resume or continue the AAD regime after the control, this was regarded as a treatment failure.

Another obvious limitation is that treatment success was only evaluated among patients residing within the hospital district of the study centre. Although the centre is the sole provider of invasive electrophysiology for a larger area (a catchment area of over million inhabitants), reliable 1-year follow-up data was available only for 63.9% of all ablation-naïve patients (patients from the Pirkanmaa Hospital District). Patients from other hospital districts (*n* = 646) were excluded from the analyses, as only 18.4% (*n* = 119) of them had follow-up data available, with a gross overrepresentation of redo patients (*n* = 67) caused by the selection bias due to referrals of only patients with obvious failures for a redo operation.

Given the observational nature of this retrospective study, we are unable to present data on all possible risk factors, such as the prevalence of sleep apnoea (not reliably captured by electronic health records) or left atrial fibrosis measurable by magnetic resonance imaging. However, we are able to present data for many other pre-existing comorbidities and patient- or hospital-related factors, and the full-disclosure review of hospital records allows full complication data to be presented with no losses to follow-up.

## Conclusions

Catheter ablation for atrial fibrillation is a relatively safe procedure with good results as regards the maintenance of sinus rhythm. Significant preoperative and early post-operative predictors for treatment success include a paroxysmal type of atrial fibrillation, left atrium size, previous use of antiarrhythmic drugs and the recurrence of atrial fibrillation during a 3-month blanking period.

## Data Availability

The data underlying this article cannot be shared publicly, since the authors are not authorised to share the data. The data will be shared upon reasonable request addressed to the corresponding author.
